# Immunogenicity of Multiple Doses of pDNA Vaccines against SARS-CoV-2

**DOI:** 10.3390/ph14010039

**Published:** 2021-01-06

**Authors:** Iman Almansour, Nabela Calamata Macadato, Thamer Alshammari

**Affiliations:** Department of Epidemic Diseases Research, Institute for Research and Medical Consultations (IRMC), Imam Abdulrahman Bin Faisal University, P.O. Box 1982, Dammam 31441, Saudi Arabia; ncmacadato@iau.edu.sa (N.C.M.); tmjalshammari@iau.edu.sa (T.A.)

**Keywords:** vaccine, SARS-CoV-2, virus, pDNA, immunity, antibody, COVID-19, coronavirus

## Abstract

Since its identification in Wuhan, China, in December 2019, severe acute respiratory syndrome coronavirus 2 (SARS-CoV-2), the causative agent of coronavirus disease 2019 (COVID-19), has resulted in 46 million cases and more than one million deaths worldwide, as of 30 October 2020. Limited data exist on the magnitude and durability of antibodies generated by natural infection with SARS-CoV-2 and whether they can provide long-lasting immunity from reinfection. Vaccination has proven the most effective measure for controlling and preventing pandemics and, thus, development of a vaccine against COVID-19 is a top priority. However, the doses required to induce effective, long-lasting antibody responses against SARS-CoV-2 remain undetermined. Here, we present the development of SARS-CoV-2 vaccine candidates encoding the viral spike (S) gene, generated using plasmid (p)DNA technology, and we demonstrate the eliciting of S-specific antibodies in mice after three and four doses. The magnitude of binding and neutralizing antibody responses with three doses of synthetic, codon-optimized, full-length S (S.opt.FL) vaccine is comparable to that generated after four doses, suggesting that three doses are sufficient to elicit robust immune responses. Conversely, four doses of S1.opt pDNA vaccine, containing the S globular head, are required to elicit high levels of neutralizing antibodies. Furthermore, the S.opt.FL pDNA vaccine induces the highest serum levels of interferon (IFN)-γ, a marker for activation of cellular immune responses. Overall, our data show that three doses of S.FL pDNA vaccine elicit potent neutralizing antibody responses, with preclinical data that support the immunogenicity of these COVID-19 vaccine candidates and provide justification for further translational studies.

## 1. Introduction

In the 21st century, three coronaviruses that have evolved the ability to cross the species barrier and infect humans have been identified: severe acute respiratory disease syndrome coronavirus (SARS-CoV), Middle East respiratory syndrome (MERS)-CoV, and, most recently, SARS-CoV-2. To date, no licensed vaccine against SARS-CoV-2 or any other human coronaviruses exists, although several SARS-CoV-2 vaccine platforms, including inactivated virus, viral vector vaccine, plasmid (p)DNA, and messenger RNA (mRNA), are being tested at various stages in clinical trials [1,2,3,4,5,6] with two mRNA vaccines being approved for emergency use [7,8].

SARS-CoV-2 entry is dependent on its surface glycoprotein, the spike (S), which binds to the angiotensin-converting enzyme 2 (ACE2) receptor on host cells [9,10,11]. The spike is a trimetric type 1 transmembrane protein, with each monomer consisting of a receptor-binding subunit (S1) and a membrane-fusion subunit S2 [12]. As with all human coronaviruses, the S protein is the primary antigenic determinant responsible for eliciting antibodies that function to prevent viral entry and fusion [13].

Human immunity against coronaviruses is primarily mediated by the production of neutralizing antibodies at levels that are sufficient to confer protection from reinfection [13]. S-specific antibodies are detected 1–2 weeks after either natural infection or vaccination [14], although the durability of these antibodies following infection with human coronaviruses varies. For example, S antibodies elicited by the endemic alpha or beta coronaviruses wane within 12 months [14,15], whereas antibodies elicited after infection with SARS-CoV or MERS-CoV can last between 12 and 36 months [13]. In the case of SARS-CoV-2, recent studies have shown that the magnitude of neutralizing antibody response is dependent on disease severity [16]. However, the persistence of these S antibodies and whether they can provide long-lasting immunity has yet to be determined. Resolving this issue is critical for vaccine development, as insufficient neutralizing antibody levels induced after immunization can present a major hurdle for generating effective immunity.

Plasmid (p)DNA vaccines offer several unique advantages. These include robust manufacturing, cost-effectiveness, and a high safety profile. Moreover, biosafety level 3 (BSL-3) facilities are not required for the generation of DNA vaccines. Previous studies on pDNA vaccines have determined that the number of doses required for effective vaccination is dependent on the antigen and virus types and how they interact with the immune system. For example, one to two doses of pDNA vaccine produce sufficient neutralizing antibodies to influenza viruses, whereas three to four doses are needed to elicit a protective immune response against human immunodeficiency virus (HIV) [17]. Here, we conducted a preclinical study to evaluate the immunogenicity of a SARS-CoV-2 pDNA vaccine containing different forms of the S gene. We designed and generated two optimized pDNA vaccine constructs encoding the full-length S and S1 domains of SARS-CoV-2 and performed a head-to-head comparison to assess their ability to induce humoral antibody-mediated responses and production of the cytokine interferon (INF)-γ in C57BL/6 mice.

## 2. Results

### 2.1. Construct Optimization and Vaccination Strategy

**Figure 1:**
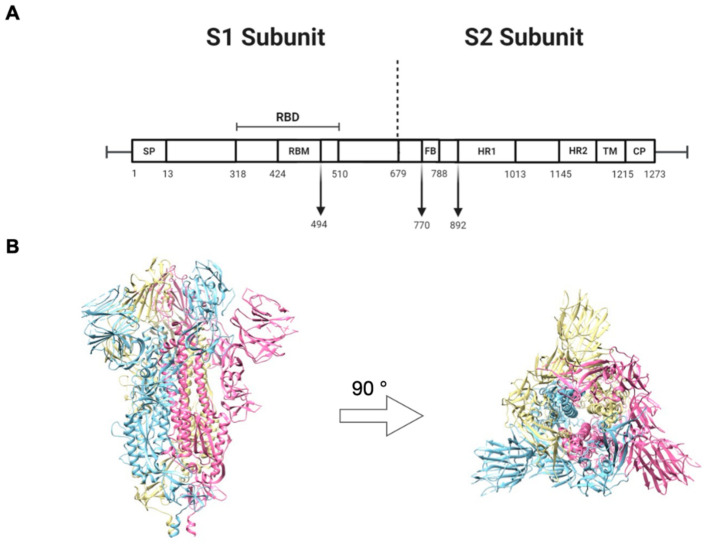
Schematic of the severe acute respiratory syndrome coronavirus 2 (SARS-CoV-2) spike glycoprotein. (**A**) The primary structure of S with its domains: signal peptide (SP), receptor binding domain (RBS), fusion peptide (FP), heptad repeat (HR), transmembrane (TM), and cytoplasmic tail (CT). (**B**) Side and top view of the three-dimensional structure of the trimeric spike protein in the perfusion confirmation. Image created from the structure with Protein Data Bank (PDB) identifier VXX6.

The S glycoprotein of SARS-CoV is composed of two subunits, S1 and S2. The S1 subunit consists of four domains, namely, the N-terminal domain (NTD), the C-terminal domain (CTD), and subdomains II and I. In addition, the S1 subunit contains the receptor-binding domain (RBD), an essential component required for binding to the human (h)ACE2 receptor on the host cell (Figure 1A). The S2 subunit consists of the fusion peptide (FP) domain, heptad repeats (HR) 1 and 2, the transmembrane domain (TM), and the cytoplasmic tail (CT). These elements are necessary for the fusion of SARS-CoV-2 with the host cell membrane (Figure 1A). The S of coronaviruses is a trimeric type I transmembrane, and each monomer consists of S1 and S2 subunits (Figure 1B).

**Figure 2:**
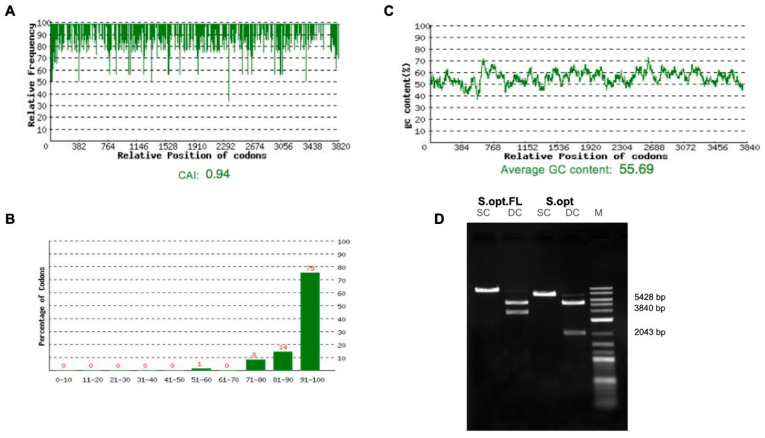
Optimizations of the full-length SARS-CoV-2 spike gene. (**A**) Distribution of codon usage frequency of the spike gene. Codon Adaptation Index (CAI) = 0.94. (**B**) Codon distribution percentage computed as codon quality group. (**C**) GC content adjustment with average equal to 55.69. (**D**) Restriction analysis for the S.opt.FL and S1.opt using single-cut digestion with *BamH*I and double-cut digestion with *BamH*I and *Nhe*I.

**Figure 3:**
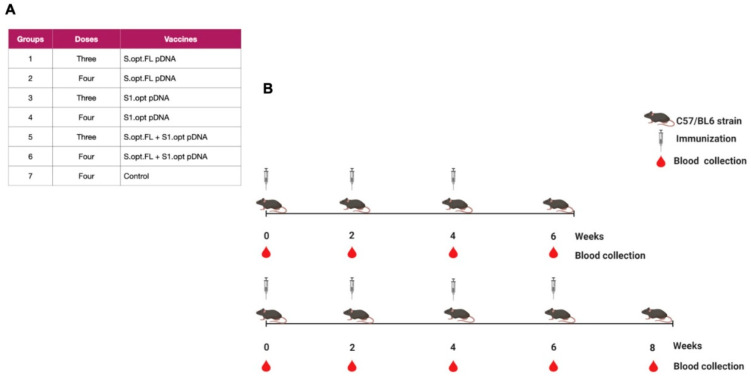
Schematic of the C57BL/6 mice immunization with SARS-CoV-2 vaccines. (**A**) Immunization groups and doses for the plasmid (p)DNA vaccines. All immunizations were received intramuscularly with 100 ug per dose, except the phosphate-buffered saline (PBS) control group. (**B**) The bleeding and immunization regime for the C57BL/6 mice.

Two vaccine constructs were tested in this study: pDNA S.opt.FL containing the full-length S gene and pDNA S1.opt including only the globular head, S1 subunit. The codons of S.FL gene were changed to mammalian codon preference (*Homo sapiens*) to enhance the gene expression in mammalian cells (Figure 2A–C) and were subsequently synthesized and inserted into pcDNA 3.1(+). Furthermore, the S1.opt was generated from S.FL via mutagenesis study. Both sequences were tested for the correct gene size (Figure 2B). Mice were divided into seven groups (*n* = 6 per group); the first group received pDNA S.opt.FL, the second group received pDNA S1.opt, and the third group received one dose of pDNA S.opt.FL followed by two doses of pDNA S1.opt. These groups each received three doses of vaccine. Group four received pDNA S.opt.FL, group five received pDNA S1.opt, and group six received one dose of pDNA S.opt.FL followed by three doses of pDNA S1.opt; these groups each received four doses of vaccine. Group seven was the control group and received only phosphate-buffered saline (PBS) (Figure 3A). A mouse from the control group died prior to first immunization and another mouse from group 4 died after first immunization.

### 2.2. Immunogenicity in Mice: Production of Binding Antibodies

**Figure 4:**
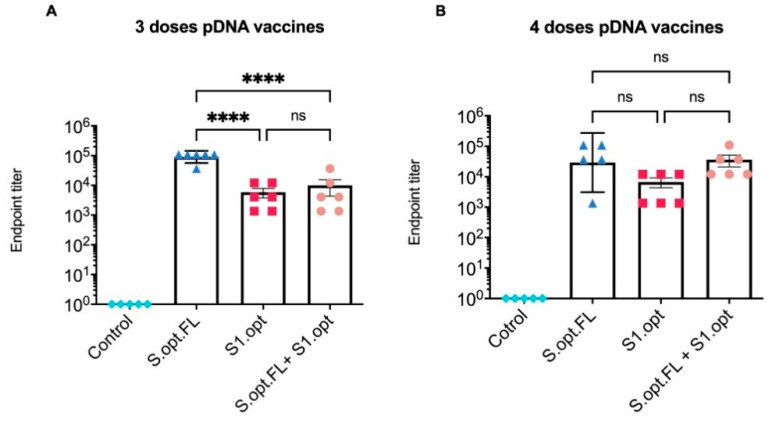
Serum endpoint immunoglobulin G (IgG) ELISA titers against autologous full-length spike (S) protein. (**A**). Total IgG S antibodies were measured in mice sera 2 weeks after the third immunization. Serum starting concentration was 1:50. (**B**) Total IgG S antibodies were measured in mice sera 2 weeks after the fourth immunization. The highest dilution that gave an optical density (OD)_450_ twofold higher than that of the prebleed sera (week 0) was designated as the antibody endpoint titer. Antibody titers were expressed as mean endpoint titers ± standard error of the mean (SEM) for each vaccine group with an individual scatter dot plot (*n* = 6). Data were compared by one-way ANOVA followed by Tukey’s multiple comparison test. ns: no significant difference. The asterisks refer to the level of significance: **** *p* < 0.0001; ns: no significant difference.

All C57BL/6 mice were vaccinated intramuscularly (IM) at 6–8 weeks of age with pDNA vaccines or the PBS control; blood was collected at 2 week intervals. Total immunoglobulin G (IgG) antibodies against the S protein were measured in serum samples collected 2 weeks after the last immunization (Figure 3B). Our results indicate that sera from all groups of immunized mice, except the PBS control group, contained detectable levels of binding antibodies at weeks 6 and 8 (Figure 4A,B). Comparisons among vaccine groups further revealed that mice vaccinated with S.opt.FL pDNA vaccine (groups 1 and 4) generated the highest levels of binding antibodies, with three and four doses of vaccine eliciting equivalent antibody responses (Figure 4A,B). Mouse groups immunized with the pDNA S1.opt vaccine produced the lowest levels of antibody responses, while the heterologous vaccine produced a moderate immune response (Figure 4A,B).

### 2.3. Immunogenicity in Mice: Production of Neutralizing Antibodies

**Figure 5:**
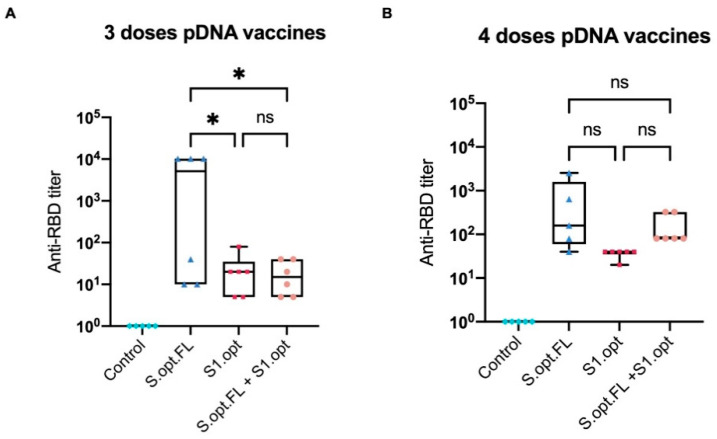
Box-and-whisker plot of surrogate virus neutralization test (sVNT). (**A**) Titer of anti-receptor-binding domain (RBD) IgG antibodies from serially diluted mice vaccinated sera taken 2 weeks after the third immunization. (**B**) Titer of anti-RBD IgG antibodies from serially diluted mice vaccinated sera taken 2 weeks after the fourth immunization. Cutoff titer was calculated as the serum highest dilution showing a cutoff value >20%. Data were analyzed with one-way ANOVA with Tukey’s multiple comparison test. The asterisks refer to the level of significance: * *p* < 0.033; ns: no significant difference.

To assess the immunological efficacy of the two pDNA vaccine candidates, a surrogate virus-neutralizing assay was performed. This technique is based on the fact that neutralizing antibodies can block the interaction between the SARS-CoV-2 RBD and the ACE2 receptor. Neutralization assay results revealed that mice who received three immunization doses with pDNA S.opt.FL produced higher levels of neutralizing antibodies than mice vaccinated with three doses of pDNA S1.opt (Figure 5A). Of note, mice immunized with S.opt.FL at weeks 6 and 8 produced similar levels of neutralizing antibodies. We further found that an additional dose enhanced the levels of neutralizing antibodies; that is, mice who received S.opt.FL priming, followed by the three S1.opt booster doses, had higher antibody responses than those who received only two S1.opt booster doses (Figure 5A,B). Interestingly, mice immunized with four doses of S1.opt produced comparable levels of neutralizing antibody responses to immunization with three doses (Figure 5A,B).

### 2.4. Immunogenicity in Mice: Production of IFN-γ

**Figure 6:**
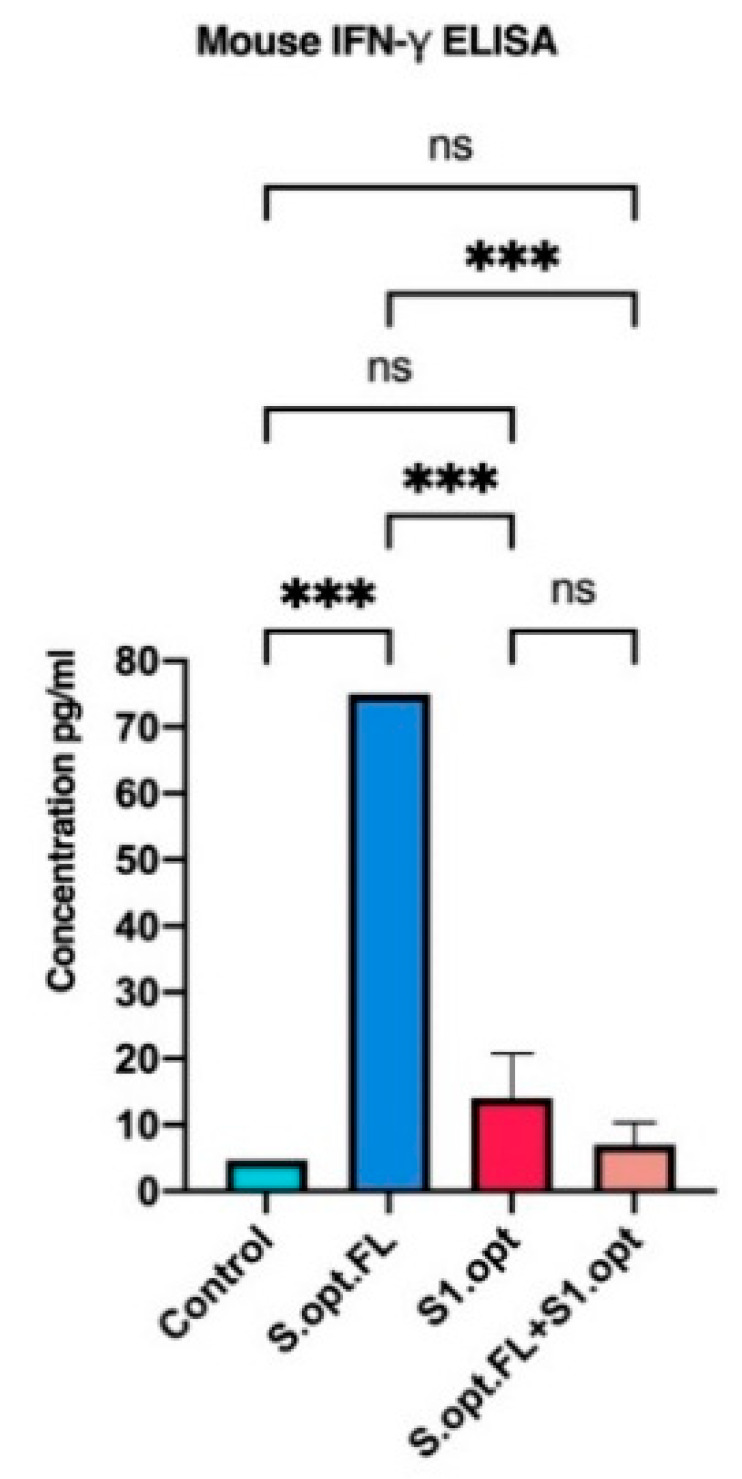
In vivo IFN-γ responses following C57BL/6 mice vaccinations. Comparing serum IFN-γ levels in each of vaccine constructs (S.opt.FL, S1.opt, and S.opt.FL+S1.opt) 2 weeks following second immunization in each vaccine construct using pooled mice sera from each group. Endpoint concentration was determined by titers expressed (mean ± SD). Data were analyzed with one-way ANOVA with Tukey’s multiple comparison test. The asterisks refer to the level of significance: *** *p* < 0.0002; ns: no significant difference.

Recent studies highlighted the role of cell-mediated responses in controlling COVID-19. We, therefore, measured the serum levels of IFN-γ in mice immunized with our vaccine constructs, as an indicator of innate immunity/cellular immunity. We found that, consistent with our antibody data, mice immunized with S.opt.FL pDNA vaccine produced significantly higher serum levels of IFN-γ, relative to the other experimental vaccine groups (Figure 6).

## 3. Discussion

The pDNA platform is as an attractive strategy for vaccine development during pandemics. This technology is simple and highly scalable. Furthermore, unlike mRNA vaccines that are fragile and require encapsulation to protect from degradation, pDNA vaccines are thermally stable, which is particularly beneficial during vaccine shipment and storage.

Limited data are available on the effect that multiple vaccine doses can have on eliciting potent neutralizing antibodies. The pDNA vaccines generated in our study encode the full-length SARS-CoV-2 S gene and S1 as the antigens of interest. In addition, combining multiple gene inserts in a plasmid vector may interfere with expression of the proteins encoded by these gene inserts; hence, we tested combined administration of the different constructs (S.opt.FL and S1.opt genes) at different doses.

Previous studies on pDNA vaccines against other viral pathogens determined that the optimal dosage required for effective immunity is dependent on the antigen/virus type and how these interact with the immune system. For example, one to two doses of pDNA vaccine are sufficient to produce effective neutralizing antibodies for influenza viruses; however, three to four doses are needed to elicit a sufficient protective immune response in HIV [17]. Neutralizing antibodies against SARS-CoV-2 target the spike RBD known to bind to ACE2 of host cell, thereby blocking viral entry [18]. However, the number of pDNA vaccine doses needed to elicit optimal neutralizing antibody responses to SARS-CoV-2 remains unexplored.

Here, we hypothesized that multiple doses of a SARS-CoV-2 pDNA vaccine would be needed to generate an effective SARS-CoV-2 antibody-mediated immune response. Therefore, we tested three and four doses of each SARS-CoV-2 pDNA vaccine to determine which of these could elicit the most potent neutralizing antibody response. Our findings show that three doses of pDNA S.opt.FL vaccine induced highest levels of neutralizing antibodies, with no added antibody production conferred by the fourth vaccine dose. In addition, the full-length S protein elicited the most potent immune response, as compared to the pDNA S1.opt vaccine or the S.opt.FL with an S1.opt booster, suggesting that multiple doses of full-length S are needed to elicit high-level immune responses. Recent bioinformatics analyses have shown that non-RBD epitopes have greater surface accessibility when compared to RBD epitopes and, thus, might be more immunogenic [18]. Some of these epitopes were located in the fusion protein (FP), heptad repeat domain 2 (HR2), and N-terminal of S2 [18]. Consistent with this observation, mice vaccinated with S.opt.FL elicited higher IFN-γ production than mice vaccinated with the S1.opt or the combined vaccine. It might be worthwhile to compare the immunogenicity of 1–2 doses of pDNA S.FL with three doses of vaccine in a further study. Furthermore, as with other newly emerging or reemerging infectious viruses [19,20,21], it will be crucial to track SARS-CoV-2 sequence variations across different locations and over time to predict any possible immune escape from the SARS-CoV-2 vaccine strain.

## 4. Materials and Methods

### 4.1. Ethics Statement

This preclinical study was registered under the Animal Study Registry 10.17590/asr.0000212. Animal protocols were approved by the Institutional Review Board (IRB NO-2020-333-IRMC) at Imam Abdulrahman Bin Faisal University (IAU), and experiments were done in compliance with the institution guidelines.

### 4.2. pDNA Vaccine Constructs

A gene construct encoding the full-length S protein (3840 bp) (YP_009724390.1) was codon-optimized for *Homo sapiens*. Additional optimizations were performed, including for GC% content, mRNA secondary structure, cryptic splicing sites, premature polyA sites, internal Chi sites, ribosomal-binding sites, and RNA stability motifs. To increase translation initiation, Kozac and Shine–Delgarno sequences were included. The S.opt.FL was de novo synthesized (GenScript, Piscataway, NJ, USA), and *Nhe*I and *BamH*I restriction sites were incorporated up- and downstream, respectively, of the coding sequence. The S.opt.FL insert was individually cloned into pcDNA 3.1(+). The nucleotide sequence of the S.opt.FL construct was confirmed by sequencing. The S1.opt construct (2043 bp) was synthesized by mutagenesis using the synthesized S.opt.FL as a template. Briefly, the mutagenesis oligo was synthesized. The S.opt.FL_pcDNA 3.1(+) was amplified by PCR using the mutagenesis oligo. The mutagenesis construct was linearized by *Nhe*I and *BamH*I and subsequently ligated. The construct was transformed into competent cells and was incubated overnight in LB media with ampicillin at 37 °C. A colony was picked and verified by colony PCR and sequencing. For pDNA vaccine production, each cloned vaccine construct was grown in LB media containing ampicillin and was incubated overnight at 37 °C. A plasmid DNA purification kit (Cat# 12163, Qiagen) was used to purify each vaccine construct. The purification levels for S.opt.FL and S1.opt, verified at absorbance 260/280, were 1.91 and 1.89, respectively. Construct lengths were checked by restriction analyses prior to immunization.

### 4.3. Immunizations

C57BL/6 mice, 6–8 weeks of age, were provided by the King Faisal Specialist Hospital and Research Center, Riyadh, Saudi Arabia. These were maintained by the animal house facility at Imam Abdulrahman Bin Faisal University. Mice were vaccinated intramuscularly (IM) into the tibialis anterior muscle. For each immunization, animals received 100 μg of pDNA in 200 μL of phosphate-buffered saline (PBS), pH 7.4, or the PBS control. Mice were administered vaccines at multiple sites. Serum samples were collected prior to the first immunization and 2 weeks after each immunization.

### 4.4. Enzyme-Linked Immunosorbent Assay (ELISA)

For ELISAs, 96-well plates (Cat# 44-2404-21; Thermo Fisher Scientific, Waltham, MA, USA) were coated with 10 ug/mL of the full-length S antigen (Cat# Z03483-1, Genscript) and incubated overnight at 4 °C. Using a 96-well plate washer, plates were washed five times with 300 µL of 1× PBS. For blocking, 200 µL of 5% non-fat dry milk in Tris-buffered saline (Blocker BLOTTO Cat# 170-6404; Bio-Rad Laboratories, Hercules, CA, USA) was added to each well, and plates were incubated for 1 h at room temperature. Blocked plates were washed five times with 300 µL of 1× PBS, and 100 µL of serially diluted serum from vaccinated mice was added to each well, followed by incubation for 1 h at room temperature. After five washes with 300 µL of 1× PBS, 100 µL of goat anti-mouse IgG secondary antibody conjugated to horseradish peroxidase (HRP) (Cat# 31430; Invitrogen, Thermo Fisher Scientific) was added to each well, and plates were incubated for 1 h at room temperature. Plates were washed five times with 300 µL of 1× PBS, and 100 µL of tetramethylbenzidine (TMB) substrate (Cat# 1854050; Thermo Fisher Scientific) was added to all wells, according to the manufacturer’s instructions. Lastly, 100 µL of 2 M sulfuric acid (2M H_2_SO_4_) was added to all wells to stop reactions; optical density (OD) values were read at 450 nm.

### 4.5. Neutralization Assay

The test used to measure antibody neutralization was based on the surrogate virus neutralization test (Cat#L0084; GenScript), a robust assay for testing vaccine efficacy. Briefly, serum samples, as well as positive and negative controls, were serially diluted and incubated with an equal volume (1:1) of diluted HRP-conjugated receptor-binding domain (RBD) at 37 °C for 30 min. Mixtures were then added to plates coated with ACE2, which were covered and incubated at 37 °C for 15 min. After washing four times with 1× wash solution, 100 µL of TMB was added to each well, and plates were incubated in the dark at room temperature for 20 min. Lastly, 50 µL of stop solution was added to each well, and the absorbance was read immediately at 450 nm. Percentage neutralization was calculated based on the following formula: (1 − sample absorbance/negative control absorbance) × 100%, with a cutoff value of >20%.

### 4.6. IFN-γ

Levels of secreted IFN-γ were measured by ELISA using the mouse IFN-γ (improved) ELISA Kit (Cat#KMC4021; Invitrogen), according to manufacturer instructions. Briefly, 100 µL of pre-diluted serum samples with standard diluent buffer were added to wells. Samples were incubated at room temperature for 2 h, and plates were washed four times with the provided wash buffer. Next, 100 μL of streptavidin–HRP solution was added to each well, and plates were incubated at room temperature for 30 min. After washing four times with wash buffer, 100 μL of stabilized chromogenic substrate was added to each well, and plates were incubated at room temperature for 30 min. Lastly, 100 μL of stopping solution was added to each well, and plates were read at 450 nm.

## 5. Conclusions

Current vaccines for SARS-CoV-2 undergoing clinical trials include those that are administered in both one dose and two doses. Here, we have shown that three doses of our SARS-CoV-2 pDNA vaccine encoding the codon-optimized, full-length S gene generated potent and robust binding and neutralizing antibodies, as well as IFN-γ cytokine responses. Thus, these results provide a valuable reference and suggest that this candidate could be moved into phase I study.

## Data Availability

The data presented in this study are available in this article.
